# Music-Making and Depression and Anxiety Before and During the COVID-19 Pandemic—Results From the NAKO Cohort Study in Germany

**DOI:** 10.3389/ijph.2024.1606993

**Published:** 2024-06-24

**Authors:** Heiko Becher, Lilian Krist, Juliane Menzel, Isabel Fernholz, Thomas Keil, Gunter Kreutz, Alexander Schmidt, Fabian Streit, Stefan N. Willich, Cornelia Weikert

**Affiliations:** ^1^ Institute of Global Health, Heidelberg University Hospital, Heidelberg, Baden-Württemberg, Germany; ^2^ Institute of Social Medicine, Epidemiology and Health Economics, Charité-Universitätsmedizin Berlin, Berlin, Germany; ^3^ Department of Food Safety, Federal Institute for Risk Assessment (BfR), Berlin, Germany; ^4^ Clinic for Audiology and Phoniatrics, Charité-Universitätsmedizin Berlin, Berlin, Germany; ^5^ Institute of Clinical Epidemiology and Biometry, Faculty of Medicine, University of Würzburg, Würzburg, Bavaria, Germany; ^6^ Bavarian Health and Food Safety Authority (LGL), Oberschleissheim, Germany; ^7^ Department of Music, Carl von Ossietzky University Oldenburg, Oldenburg, Lower Saxony, Germany; ^8^ Department of Genetic Epidemiology in Psychiatry, Department of Psychiatry and Psychotherapy, Hector Institute for Artificial Intelligence in Psychiatry, Central Institute of Mental Health, Medical Faculty Mannheim, University of Heidelberg, Mannheim, Germany

**Keywords:** music making, mental health, epidemiologic study, COVID-19, cohort

## Abstract

**Objectives:**

To investigate the association of musical activity with mental health during the COVID-19 pandemic.

**Methods:**

A total of 3,666 participants reported their musical activity before and mental health indicators before and during the pandemic. Depression was assessed with the Patient Health Questionnaire, anxiety with the Generalized Anxiety Disorder scale. The association between mental health scores and musical activities was investigated using linear regression.

**Results:**

Within the last 12 months, 22.1% of the participants reported musical activity (15.1% singing, 14.5% playing an instrument). Individuals with frequent singing as their main musical activity had higher scores before the pandemic than non-musicians and the worsening during the pandemic was more pronounced compared to non-musicians. Instrumentalists tended to have slightly lower scores than non-musicians indicating a possible beneficial effect of playing an instrument on mental health.

**Conclusion:**

The pandemic led to a worsening of mental health, with singers being particularly affected. Singers showed poorer mental health before the pandemic. The tendency for instrumentalists to report lower depression scores compared to non-musicians may support the hypothesis that music-making has a beneficial effect on health.

## Introduction

During the first wave of the COVID-19 pandemic in 2020, a series of corona contact restriction measures were introduced in Germany to reduce the rapidly increasing number of infections. This led to a so-called lock-down from March 22 to 5 May 2020, including closure of public spaces, businesses, and educational and cultural institutions [1]. The measures inevitably led to more social isolation for many individuals in the general population [2].

There is sufficient evidence that social isolation in general increases the risk of physical and mental illness as well as mortality [3–7]. This has been confirmed in the context of COVID-19 in various settings [8], and also in Germany within the German National Cohort study (NAKO Gesundheitsstudie) [9]. Here, an increase in loneliness during the first wave of the COVID-19 pandemic in spring 2020 and a strong relationship associating increasing loneliness with decreasing mental health has been observed [10].

Several studies have shown that activities such as making music are associated with lower levels of biological stress in daily life and lower anxiety [11]. Singing and music-making in the amateur sector, for example, were found to be linked to positive effects on both mental and physical health [12–14], and amateur musicians often report an increased sense of wellbeing due to their regular participation in musical activities [15].

The effect of musical activities on physical and mental health during the COVID-19 pandemic has been the subject of a wide range of studies. On the one hand, playing musical instruments such as woodwind or brass instruments in ensembles or choir singing in enclosed spaces has been identified as a possible source of virus transmission via the spread of aerosols [16, 17]. On the other hand, the same activities are associated with beneficial effects on mental health and wellbeing. In one cross sectional study, musical activity (listening to, playing, singing, etc.) was identified as a coping mechanism for psychological distress during the pandemic and people making music showed less depressive symptoms [18]. In a study performed in Spain with 1868 Spanish citizens, musical engagement (listening, singing, dancing or playing an instrument) has been associated with less symptoms of anxiety and increased subjective wellbeing in times of COVID-19 [19]. Musical activity and other creative arts were identified as a tool to cope with stressful situations during the pandemic [20]. Different age groups seem to benefit from musical activity in different ways. While younger people rated musical activities most effective as a coping mechanism during pandemic lockdowns, older people (55+) reported non-musical activities to be more helpful [21].

The present study aims to investigate whether the accumulated hours of musical activities over the lifetime as well as over the past 12 months affected the self-reported mental health status at different time points before and during the COVID-19 pandemic in amateur singers and instrumentalists. To achieve this, we investigate one cohort that was examined using two different measures of individual mental health before and during the COVID-19 pandemic, respectively.

## Methods

The German National Cohort (NAKO) is a large prospective cohort study with some 205,000 participants conducted in 18 study centers which are distributed all over Germany [22]. The baseline examination took place from 2014 to 2019. All study participants underwent an extensive examination program and a detailed interview including demographic variables, lifestyle factors and extensive data on health issues. Regarding mental health, the Patient Health Questionnaire PHQ-9 (a diagnostic tool to screen for depression [23] which has a range from 0 to 27 and denotes the severity of depressive symptoms with a score of 0–4 no symptoms, 5–9 mild, 10–14 moderate, 15–19 moderately severe, and 20–27 severe symptoms), and the Generalized Anxiety Disorder (GAD-7) scale (a questionnaire for screening of generalized anxiety disorder [24] which yields scores between 0 and 21 where 0–5 represent mild, 6–10 moderate, 11–14 moderately severe anxiety, and 15–21 severe anxiety) were assessed. Between May and July 2020, the first year of the pandemic, all NAKO participants were re-contacted and 161,849 study participants answered a COVID-questionnaire asking about pandemic related issues and in which PHQ-9 and GAD-7 were re-assessed.

Since 2017 in the NAKO study center Berlin-Mitte (Berlin Central). An additional questionnaire has been applied to assess active and receptive music activities [25]. Type of instrument, frequency of musical activities in different phases of life starting in childhood were assessed. In addition, musical activity within the last 12 months was assessed. 11,048 individuals were recruited in Berlin-Mitte for the NAKO baseline examination. Of these, 3,691 participants completed the music questionnaire before the start of the COVID-19 lockdown in Germany, i.e., before March 2020, and participated in the additional survey during the pandemic in 2020. There were 25 professional musicians (15 males, 10 females) in the study population (ISCO (International standard Classification of Occupations) code 2,652 or KLDB (Klassifikation der Berufe “Classification of occupations”) code 94114–94184) [26, 27]. Since we only focused on amateur musicians in this paper, these 25 professional musicians were excluded from the analyses. The following analysis is thus based on N = 3,666 individuals (1830 males, 1836 females) from the NAKO study center Berlin-Mitte.

We first present some descriptive results on demographic characteristics, mental health scores at baseline and during the pandemic, as well as musical activities as assessed before the pandemic.

We then model the potential effect of the reported musical activity (only assessed before the pandemic) on mental health (assessed both before and within the pandemic) with a linear regression model. The individual PHQ-9 and GAD-7 scores in the baseline examination, i.e., before the pandemic, as well as the individual differences of the PHQ-9 and GAD-7 scores between assessment at baseline and during the pandemic were used as dependent variables. When modelling the differences, the initial value of the score was included as covariable in the model. We performed an adjustment for sex and age (continuous), and additionally for the following demographic and lifestyle factors: education (higher (ISCED level >=5 vs. lower (ISCED level 1–4), migrant status (yes (either parent or self migrated to Germany vs. no migration background), living alone (living with partner/spouse vs. living alone/widowed) current smoking (yes/no), obesity (BMI>30, yes/no), risky alcohol consumption ((AUDIT-C Score >4 in Males, >3 in females) yes/no). The model was motivated by a DAG and limited by the variables that were available in the dataset [28]. The above variables have been found to be associated with mental health before, and the music variables were the main topic of this paper. A formal modelling procedure with e.g., backward selection has not been performed. To descriptively assess the degree of confounding of the covariables with the music variables, we also fitted the model only adjusted for sex and age. We also investigated possible differential effects of sex and age.

Musical activity was operationalized as follows: For each decade of life, the number of years and the average number of hours per week of musical activity were surveyed. From this information, (i) the cumulative total hours and (ii) the average duration per year of musical activity were calculated. For the latter, we divided the cumulative total hours by the reported years of musical activity. Musical activity within the last 12 months was indicated as music-hours within the last 12 months. To distinguish between the mode and intensity of musical activity (singing and/or playing an instrument) within the last 12 months, we formed three categories as follows: frequent (four times per week or more)/low to moderate (more than once a month and up to three times per week)/no or very low (up to once a month).

## Results

Some basic characteristics of the study group are given in Table 1. Information on musical activities is given in Table 2. Mean ages were 50.6 and 50.4 years in males and females, respectively, with a range from 20 to 70 years for both sexes.

**TABLE 1:** Distribution of socio-demographic and lifestyle variables in the study population study on musical activity and mental health scores [German National Cohort, Berlin, Germany, 2017–2020].

**Variable**		Males	Females	Total
		N (%)	N (%)	
**Age group (years)**	**20 − <30**	170 (9.29)	180 (9.80)	350
	**30 − <40**	166 (9.07)	183 (9.97)	349
	**40 − <50**	472 (25.79)	449 (24.46)	921
	**50 − <60**	483 (26.39)	509 (27.72)	992
	**60 +**	539 (29.45)	515 (28.05)	1,054
**Obesity** ^a^	**No**	1,441 (78.74)	1,486 (80.94)	2,927
	**Yes**	389 (21.26)	350 (19.06)	739
**Current Smoker**	**No**	1,429 (78.09)	1,434 (78.10)	2,863
	**Yes**	401 (21.91)	402 (21.90)	803
**Risky alcohol consumption** ^b^	**No**	1,053 (57.54)	1,203 (65.52)	2,256
	**Yes**	777 (42.46)	633 (34.48)	1,410
**Living alone**	**No**	1,365 (74.59)	1,340 (72.98)	2,705
	**Yes**	465 (25.41)	496 (27.02)	961
**Educational level** ^c^	**Low/medium**	678 (37.05)	740 (40.31)	1,418
	**high**	1,152 (62.95)	1,096 (59.69)	2,248
**Migrant status**	**No**	1562 (85.36)	1,550 (84.42)	3,112
	**Yes**	268 (14.64)	286 (15.58)	554
**Total**		1830	1836	3,666

^a^ Body mass index >30 kg/m2.

^b^ AUDIT-C (Alcohol Use Disorders Identification Test) Score >4 in Males, >3 in females.

^c^ ISCED 97-Level (International Standard Classification of Education).

**TABLE 2:** Lifetime and recent musical activity by sex of 3,666 German National Cohort participants included in the evaluation of musical activity and mental health [German National Cohort, Berlin, Germany, 2017–2020].

		Lifetime hours (h) of music making						
		0	>0–500 h	>500–1,000 h	>1,000–2000 h	>2000–5,000 h	>5,000 h	Total
**Males**	N	1,078	221	141	132	164	94	1830
	%	58.91	12.08	7.70	7.21	8.96	5.13	
	age^a^	52.4	48.2	47.1	47.8	48.6	50.9	
**Females**	N	963	258	178	182	184	71	1836
	%	52.45	14.05	9.69	9.91	10.02	3.87	
	age	52.5	49.3	50.0	45.5	46.3	51.0	
**Total**	N	2041	479	319	314	348	165	3,666
	%	55.67	13.07	8.70	8.57	9.49	4.50	
		**Hours per year of music making**						
				**0**	**>0–100** ** ** **h**	**>100–200** ** ** **h**	**>200–500** ** ** **h**	**>500–1,000** ** ** **h**	**>1,000** ** ** **h**	**Total**
**Males**	N	1,078	134	310	260	44	4	1830
	%	58.91	7.32	16.94	14.21	2.40	0.22	
	age	52.4	48.1	47.4	49.3	50.2	50.0	
**Females**	N	963	173	433	237	27	3	1836
	%	52.45	9.42	23.58	12.91	1.47	0.16	
	age	52.5	49.5	48.5	46.3	49.4	45.0	
**Total**	N	2041	307	743	497	71	7	3,666
	%	55.67	8.37	20.27	13.56	1.94	0.19	
		**Hours of music making within last 12** **months**						
				**0**	**>0–100** ** ** **h**	**>100–200** ** ** **h**	**>200–500** **h**	**>500–1,000** ** ** **h**	**>1,000** ** ** **h**	**Total**
**Males**	N	1,479	162	88	68	23	10	1830
	%	80.82	8.85	4.81	3.72	1.26	0.55	
	age	51.4	45.6	49.1	50.3	50.2	50.0	
**Females**	N	1,386	225	133	70	12	10	1836
	%	75.49	12.25	7.24	3.81	0.65	0.54	
	age	51.6	46.6	46.6	47.0	45.0	52.0	
**Total**	N	2,865	387	221	138	35	20	3,666
	%	78.15	10.56	6.03	3.76	0.95	0.55	

^a^ mean age within groups.

Of the whole sample, 55.7% reported no musical activities in their life (58.9% of males, 52.5% of females) which corresponds to 44.3% who reported any musical activity in their life (41.1% of males, 47.5% of females). Table 2 shows the percentages for the five different activity levels. The corresponding percentage for no musical activity within the 12 months before interview were considerably higher (78.2 total, 80.8% males, 75.5% females), since many individuals quit making music at adult age. Table 2 also includes the mean ages in the respective categories and shows that there is relatively little variation by categories of musical activity.

Table 3 presents the mean mental scores, their standard deviations and the number of individuals by musical activity before and during the pandemic and the resulting differences by sex. Again, we observe no clear trend with musical activity. No distinction is made between singing and playing an instrument.

**TABLE 3:** Mean depression (PHQ-9) and anxiety (GAD-7) score by sex and frequency groups of musical activity within the preceding 12 months [German National Cohort, Berlin, Germany, 2017–2020].

	Musical activity hours last 12 months	PHQ-9^a^							GAD-7^a^						
			Baseline (before 2020)		In 2020		Difference			Baseline (before 2020)		In 2020		Difference	
		n	$\bar{x}$	s.e.	$\bar{x}$	s.e.	$\bar{x}$	s.e.	n	$\bar{x}$	s.e.	$\bar{x}$	s.e.	$\bar{x}$	s.e.
**Males**	0	1,205	3.36	0.10	3.90	3.36	0.53	0.10	1,194	2.59	0.08	2.94	0.10	0.35	0.09
	>0–100	143	4.15	0.33	4.30	4.15	0.15	0.38	141	3.01	0.22	3.20	0.25	0.19	0.24
	>100–200	73	3.68	0.35	4.08	3.68	0.40	0.37	74	2.92	0.25	3.36	0.35	0.45	0.30
	>200–500	57	3.26	0.47	3.42	3.26	0.16	0.35	57	3.04	0.40	2.86	0.39	−0.18	0.22
	>500	26	2.96	0.79	3.42	2.96	0.46	0.63	26	3.00	0.81	3.31	0.72	0.31	0.54
	Total	1,504	3.44	0.09	3.92	0.10	0.48	0.09	1,492	2.67	0.07	2.99	0.09	0.32	0.07
**Females**	0	1,112	4.05	0.11	4.56	0.12	0.50	0.12	1,116	3.29	0.09	3.71	0.11	0.42	0.10
	>0–100	197	3.94	0.22	5.07	0.29	1.13	0.28	196	3.60	0.21	4.35	0.24	0.74	0.25
	>100–200	117	4.27	0.31	5.51	0.40	1.24	0.42	114	3.74	0.31	4.78	0.39	1.04	0.43
	>200–500	58	4.97	0.58	5.71	0.57	0.74	0.50	56	3.95	0.46	4.57	0.47	0.63	0.47
	>500	18	5.50	1.33	5.17	1.04	−0.33	1.63	16	4.63	0.89	4.75	1.25	0.13	1.59
	Total	1,502	4.11	0.09	4.75	0.10	0.64	0.10	1,498	3.40	0.08	3.92	0.09	0.51	0.09
**total**		3,006	3.77	0.07	4.32	0.07	0.56	0.07	2,990	3.04	0.05	3.45	0.06	0.42	0.06

n, number of participants.

$\bar{x}$ , mean score.

s.e., standard error.

^a^ PHQ-9 Range 0–27; GAD-7 Range 0–21.

Information on PHQ-9, and GAD-7 either at baseline or at the COVID-questionnaire were missing for 660 and 676 individuals, respectively. The mean (standard error) PHQ-9 scores at baseline and in 2020 are 3.77 (0.07) and 4.32 (0.07), and the respective mean GAD-7 scores are 3.04 (0.05) and 3.45 (0.06). We observe a clear increase in 2020, i.e., during the COVID pandemic, compared to baseline, as already shown for the total NAKO study [9]. The percentage of individuals with moderately severe to severe symptoms of depression and with moderate or serious symptoms of anxiety disorder were 7.4% and 4.6%, respectively, at baseline, and 9.0% and 5.1%, respectively, in the re-examination.

**FIGURE 1:**
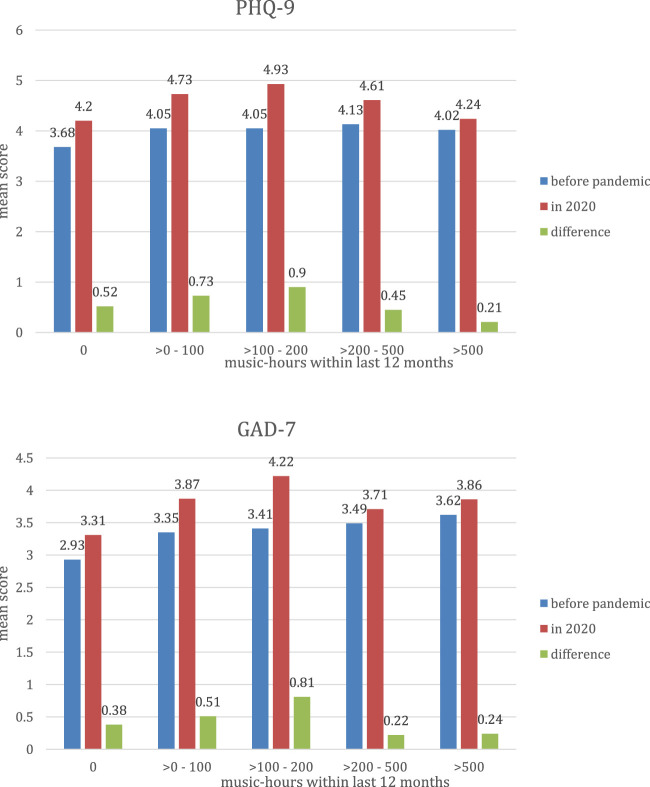
Means of the mental health scores PHQ-9 and GAD-7 and differences, stratified by categories of music-hours within last 12 months among participants from the German National Cohort study center in central Berlin [German National Cohort, Berlin, Germany, 2017–2020].

Figure 1 shows the means of the mental health scores in the baseline and in the re-examination, i.e., before and during the pandemic, and their differences, stratified by sex and categories of music-hours within last 12 months. For individuals without musical activity as well as for all groups of musical intensity there is a mean increase of the mental scores. There is no clear trend, however, we observe a less pronounced worsening of the mental scores within the two groups with highest musical activity.

In a further analysis we distinguished between singing and playing an instrument, as we hypothesize a differential effect. In Table 4 we provide the mean PHQ-9 and GAD-7 scores at baseline (before the pandemic) and in 2020 (during the pandemic), and the difference between both, for three groups of musical activity (no activity, up to three times per week, more than three times per week). We observe a higher score of both PHQ-9 and GAD-7 in frequent singers at baseline and a more pronounced worsening of the scores at the pandemic. This did not appear to be the case for instrumentalists. Here the worsening is less pronounced in comparison with non-musicians, although the difference is small. For example, the mean PHQ-9 score in non-singers before and during the pandemic was 3.70 and 4.24, with a difference of 0.55. In frequent singers the corresponding values were higher at 4.74 and 6.08, with a higher difference of 1.25. There are a few individuals which reported both singing and playing an instrument as musical activity, however the numbers were too small to allow a separate analysis of this group.

**TABLE 4:** Mean depression (PHQ-9) and anxiety (GAD-7) score by frequency groups of singing or playing an instrument within the last 12 months [German National Cohort, Berlin, Germany, 2017–2020].

Main musical activity		PHQ-9^a^ (depression)				GAD-7^a^ (anxiety)			
			Baseline	In 2020	Difference		Baseline	In 2020	Difference
		n	$\bar{x}$ (s.e.)	$\bar{x}$ (s.e.)	$\bar{x}$ (s.e.)	n	$\bar{x}$ (s.e.)	$\bar{x}$ (s.e.)	$\bar{x}$ (s.e.)
	No	2,548	3.70 (0.07)	4.24 (0.08)	0.55 (0.07)	2,599	2.98 (0.06)	3.35 (0.07)	0.37 (0.06)
**Singing**	Up to 3 times per week	296	4.03 (0.21)	4.52 (0.23)	0.48 (0.24)	299	3.20 (0.16)	3.79 (0.18)	0.60 (0.19)
	4 times per week or more	89	4.74 (0.44)	6.08 (0.50)	1.25 (0.50)	92	4.10 (0.40)	5.16 (0.42)	1.07 (0.45)
	No	2,645	3.77 (0.07)	4.30 (0.08)	0.54 (0.07)	2,699	3.01 (0.06)	3.42 (0.07)	0.41 (0.41)
**Playing an instrument**	Up to 3 times per week	223	3.85 (0.22)	4.70 (0.25)	0.89 (0.26)	226	3.27 (0.20)	3.77 (0.22)	0.50 (0.50)
	4 times per week or more	65	3.45 (0.47)	3.86 (0.47)	0.42 (0.49)	65	3.32 (.45)	3.65 (0.47)	0.32 (0.32)

Note:

*n*, number of participants;

$\bar{x}$ , mean score (lower values indicate better mental health)

s.e., standard error

^a^ PHQ-9 Range 0–27; GAD-7 Range 0–21.

In the linear regression analysis, we investigated the association of musical activity (separately for singing and playing an instrument) with the depression and anxiety symptom scores adjusted for possible confounding factors. As possible confounding factors we considered age, sex, obesity, smoking, alcohol consumption, education, living conditions (living alone) and migrant status. We analysed both the baseline score as well as the change from baseline to pandemic as dependent variable.

Amateur singers with high singing frequency have higher baseline PHQ-9 and GAD-7 scores, respectively, compared to non-singers. The estimated level is 0.83 points higher for PHQ-9 (*p* = 0.02, 95% CI 0.13–1.54) and 0.80 points higher (*p* < 0.01, 95% CI 0.21–1.40) for GAD-7 compared to non-singers. For both scores, amateur instrument players (high frequency) show no differences compared to non-musicians at baseline (0.30 points lower for PHQ-9 (*p* = 0.49, 95% CI −1.16–0.55) and 0.25 points higher (*p* = 0.49, 95% CI −0.47–0.98) for GAD-7. The scores decrease with age and are increased for females compared to males, for obese individuals, for smokers, for individuals living alone, and for migrants. The results are similar for both mental scores with the exception of education, where a lower level is associated with a high PHQ-9 score but not with the GAD-7 score (Table 5). The estimates for musical activity are almost unchanged in a model without adjustment for other covariables, indicating little confounding (data not shown).

**TABLE 5:** The association of recent musical activities and other variables with depression (PHQ-9) and anxiety (GAD-7) at baseline (before COVID-19) and on individual change of both scores at COVID-19 pandemic, 2020, multivariable linear regression analysis [German National Cohort, Berlin, Germany, 2017–2020].

		Dependent variable							
		Score at baseline				Score change from baseline to pandemic			
		Depression (PHQ-9)		Anxiety (GAD-7)		Depression (PHQ-9)		Anxiety (GAD-7)	
**Independent Variables**		**β**	**95% CI** ^b^	**β**	**95% CI**	**β**	**95% CI**	**β**	**95% CI**
**Intercept**		2.49	2.03–2.95	1.67	1.29–2.06	1.58	1.10–2.06	1.08	0.66–1.49
**Score at baseline**						−0.45	−0.49–−0.42	−0.43	−0.47–−0.39
**Sex**	female vs. male	0.65	0.40–0.89	0.74	0.53–0.95	0.42	0.17–0.67	0.44	0.22–0.66
**Age** ^**a**^		−0.03	−0.04–−0.02	−0.02	−0.03–−0.01	−0.04	−0.05–−0.03	−0.04	−0.05–−0.03
**Playing an instrument, last 12 months**	Medium^c^ vs. no	0.13	−0.34–.61	0.15	−0.25–0.55	0.21	−0.27–0.69	−0.05	−0.47–0.36
	frequent^c^ vs. no	−0.30	−1.16–0.55	0.25	−0.47–0.98	−0.25	−1.10–0.60	−0.04	−0.79–0.70
**Singing, last 12 months**	medium^c^ vs. no	0.12	−0.30–0.55	−0.10	−0.45–0.26	−0.09	−0.51–0.33	0.11	−0.26–0.48
	frequent^c^ vs. no	0.83	0.13–1.54	0.80	0.21–1.40	0.70	−0.04–1.44	0.78	0.14–1.42
**Obese**	yes vs. no	0.69	0.38–1.00	0.27	0.01–0.54	0.09	−0.22–0.41	−0.15	−0.42–0.12
**Smoker**	yes vs. no	0.70	0.40–1.00	0.49	0.24–0.74	−0.09	−0.39–0.22	−0.17	−0.44 - 0.09
**Alcohol consumption**	yes vs. no	0.04	−0.21 - 0.29	0.17	−0.04 - 0.38	0.10	−0.15 - 0.36	−0.06	−0.28 - 0.16
**Living alone**	yes vs. no	1.01	0.72–−1.28	0.35	0.12–0.59	0.32	0.04–0.61	0.11	−0.13 - 0.40
**Education**	high vs. low	−0.43	−0.68–−0.17	−0.11	−0.32 - 0.10	−0.13	−0.39–0.13	0.03	−0.19 - 0.26
**Migrant**	yes vs. no	0.95	0.60–1.30	0.68	0.39–0.98	0.14	−0.22–0.51	0.08	−0.23 - 0.39

^a^ Scaled as age-50, such that the intercept refers to score resp. score change at age 50.

^b^ CI, Confidence interval.

^c^ medium—up to 3 times per week; frequent—4 times per week or more.

The analysis of the change of the depression and anxiety symptom scores are shown in the right columns of Table 5. Overall, the scores are higher during the pandemic, indicating a negative effect of the pandemic on mental health (1.58, *p* < 0.0001, 95% CI 1.10–2.06 in PHQ-9 and 1.08, *p* < 0.001, 95% CI 0.66–1.49 in GAD-7). The worsening from baseline to the time of the pandemic in both scales, PHQ-9 and GAD-7, is stronger for females vs. males and for young ages vs. older age. Singing with high frequency before the pandemic was associated with a more pronounced worsening of the mental scores [0.69 for PHQ-9 (*p* = 0.06, 95% CI −0.04–1.44) and 0.78 for GAD-7 (*p* = 0.02, 95% CI 0.14–1.42)]. Frequently playing an instrument showed negative regression coefficients indicating a less pronounced worsening (−0.25 for PHQ-9, *p* = 0.56, 95% CI −1.10–0.59 and −0.04 for GAD-7, *p* = 0.91, 95% CI −0.79–0.70), however this estimate has a large variation and a wide confidence interval. The other factors showed little association, except living alone for which we observe a worsening of the depression score (0.32 for PHQ-9, *p* = 0.03, 95% CI 0.04–0.61).

In a further analysis we investigated the association of lifetime musical activities with the mental scores before the pandemic and the change of the scores during the pandemic. Neither the lifetime music-hours nor the music-hours per year of music making had an effect on either the baseline score or the score change with regression estimates close to zero for all models.

## Discussion

The study was designed to address potential associations between musical activities of non-professional singers and instrumentalists with their self-reported mental health status at different time points before and during the COVID-19 pandemic. We expected beneficial effects of musical activity for instrumentalists during the pandemic, however were concerned about the choir ban and following effects on mental health in singers. Results indicate that frequent singers, as compared to non-singers, had higher anxiety and depression scores even at baseline and had a stronger deterioration in both scales than non-singers during the pandemic. In instrumentalists, we found no relevant differences in the anxiety and depression scores compared to non-instrumentalists, neither in the baseline comparison nor in the development during the pandemic. In particular, our observations regarding the higher mental health scores in very active singers before the pandemic stand in contrast to earlier findings [11] although another recent study reports a similar effect [29].

The finding on the worsening during the pandemic, however, can be considered as plausible [30]. Singing was considered to be hazardous due to the increased likelihood of virus transmission, and singing in a choir was not allowed at the time of the COVID survey. However, unfortunately, no data on musical activity during the pandemic are available. Interestingly, the worsening of the mental scores in singers was even higher among those who live alone. This may lead to the assumption that for these individuals singing was a tool to foster social interaction which was reduced during the pandemic. We assume that it may have had an impact on the mental wellbeing of amateur musicians whether or not they were able to make music during the pandemic. The pandemic-related restrictions for music ensembles, e.g., due to lockdown measures or prohibition of group rehearsals, limited the musical activities of amateur choir singers and those of amateur orchestra and chamber musicians. Pandemic-related reduction of musical activity may have been smaller for piano players or other musicians who play an instrument on their own [31]. It was, however, not possible to further disentangle these groups, and the resulting estimate did not show a clear effect on mental health of instrumentalists. There is some tendency that frequently playing an instrument helped to cope better with the pandemic.

There is a strong sex effect in our data, both in terms of baseline PHQ-9 and GAD-7 score as well as in terms of coping with conditions during the pandemic. In both terms females had higher scores compared to males. There is, however, no differential effect of musical activities in both sexes, although admittedly the power to detect an interaction is low. The same holds for age. Older people tended to have better mental health, and coped better with the pandemic. Again, no interaction with musical activity was found.

Another aspect refers to a possible gene interaction between depression and musical activity. In a study of Wesseldijk et al. it was shown that individuals with higher polygenic scores for major depression and bipolar disorder were more likely to play music, practice more music and reach higher levels of general artistic achievements, while a higher genetic propensity for general musicality was marginally associated with a higher risk for a depression diagnosis [32]. Since blood samples are available from the study for further genetic analyses, this could be a topic for future investigations.

There are some limitations with the study. This is an observational study which does not allow causal effects to be assessed. We also acknowledge that a change of musical activities has not been assessed. In addition, a non-participant bias cannot be excluded. This holds primarily for the analysis in the mental score at baseline. It is not unlikely that individuals with depression or anxiety have a lower likelihood of participating in an epidemiological study as volunteers, and this would lead to an underestimation of the mental disease prevalence. We also observe a higher response in individuals with higher education. School education is correlated with musical activities, and therefore the prevalence of these may also be overestimated. However, we consider it less likely that there is a differential response for musical activities given education, and this would allow an unbiased effect estimate of musical activities on mental health scores We note that the results of the linear regression models are not suitable to predict the PHQ-9 or the GAD-7 score but only to explain the relevance of some of the variables.

In conclusion, our results suggest worsening of mental health during the pandemic in particular for singers, which was not unexpected due to the contact restrictions. Furthermore, we observed tendencies of less pronounced worsening of depression scores for instrumentalists, which may indicate beneficial health effect of making music.
